# Association of the Red Cell Distribution Width–Albumin Ratio with Myocardial Strain, Mortality, and Heart Failure Hospitalization: A Retrospective Cohort Study

**DOI:** 10.3390/jpm16100530

**Published:** 2026-10-14

**Authors:** Sherif Ahmed, Kamal Awad, Abdelrahman Hafez, Juan M. Farina, Mohamed Nour, Isabel G. Scalia, Fatmaelzahraa Abdelfattah, Laureve Chollet, Omar Seleem, Mahshad Razaghi, Said Alsidawi, Steven J. Lester, David Simper, Chadi Ayoub, Reza Arsanjani

**Affiliations:** 1 Department of Cardiovascular Medicine, Mayo Clinic Arizona, 5777 E Mayo Blvd, Phoenix, AZ 85054, USA; ahmed.sherif@mayo.edu (S.A.); awad.kamal@mayo.edu (K.A.); abdelrahmanafez13498@gmail.com (A.H.); juan.farina@gmail.com (J.M.F.); nour.mohamed@mayo.edu (M.N.); scalia.isabel@mayo.edu (I.G.S.); abdelfattah.fatmaelzahraa@mayo.edu (F.A.); chollet.laureve@mayo.edu (L.C.); seleem.omar@mayo.edu (O.S.); razaghi.mahshad@mayo.edu (M.R.); alsidawi.said@mayo.edu (S.A.); lester.steven@mayo.edu (S.J.L.); simper.david@mayo.edu (D.S.); ayoub.chadi@mayo.edu (C.A.); 2 Faculty of Medicine, The University of Queensland, Brisbane, QLD 4006, Australia; 3 Cardiovascular Research Institute Basel (CRIB) and Department of Cardiology, University Hospital of Basel, University of Basel, 4056 Basel, Switzerland

**Keywords:** red cell distribution width–albumin ratio (RDW/Albumin), left ventricular global longitudinal strain (LVGLS) impairment, heart failure hospitalization risk, all-cause mortality risk, subclinical myocardial dysfunction, cardiovascular biomarkers

## Abstract

**Aims:** The aims of this study were to evaluate the association between the red cell distribution width (RDW)–albumin ratio (RAR) and subclinical myocardial impairment, as assessed by left ventricular global longitudinal strain (LVGLS), and to examine the relationships between the RAR, heart failure (HF) hospitalization, and all-cause mortality. **Methods:** We conducted a retrospective cohort study on adults who underwent transthoracic echocardiography (TTE) with LVGLS assessment across three Mayo Clinic centers (2017–2024) and who had RDW and serum albumin measured on the same day or within seven days. The RAR was calculated as RDW/albumin and categorized into the following tertiles: <2.93, 2.93–3.48, and >3.48. Impaired LVGLS was defined as >−18%. Associations between the RAR and outcomes were assessed using multivariable logistic regression (LVGLS) and Cox models (HF and mortality), adjusting for demographics and comorbidities. **Results:** Among 5069 patients, 39.7% had impaired LVGLS, and its prevalence increased across RAR tertiles (26.8%, 41.9%, and 51.1%). Adjusted odds ratios for impaired LVGLS were 1.74 and 2.30 for tertiles 2 and 3, respectively. A higher RAR was associated with increased risk of HF hospitalization (aHR 2.54 and 4.48) and all-cause mortality (aHR 2.39 and 7.30). These associations persisted after adjusting for LVGLS. The effect of the RAR on mortality was stronger in patients with normal LVGLS compared to those with impaired LVGLS (*p* for interaction = 0.0004). **Conclusions:** A higher RAR was associated with abnormal LVGLS, HF hospitalization, and all-cause mortality after adjustment for clinical risk factors. However, the RAR did not provide discriminative value beyond its individual components.

## 1. Introduction

The red cell distribution width (RDW) is a readily available laboratory measurement used to assess the variability of red blood cell sizes. It has been linked to adverse cardiovascular events such as heart failure (HF) and mortality [1]. Albumin is another routinely measured biomarker that is used to assess nutritional status and serves as a negative acute phase reactant reflecting inflammatory status [2]. Like the RDW, low albumin has also been linked to adverse cardiovascular outcomes and poorer prognoses. In light of the progressive increase in both the incidence and mortality associated with cardiovascular disease (CVD), the identification of a rapid, cost-effective, and non-invasive biomarker for the recognition of high-risk individuals is important to implement preventive strategies to decrease the CVD burden [3]. The RDW–albumin ratio (RAR) combines both RDW and albumin, integrating hematologic, inflammatory, and nutritional information into a single marker that has been shown to be associated with worse outcomes in various clinical settings [4].

However, the mechanisms underlying these associations are still not well-defined. One potential pathway involves subclinical myocardial dysfunction, which may precede overt structural changes. In this context, left ventricular global longitudinal strain (LVGLS) is a sensitive echocardiographic measurement denoting subclinical myocardial impairment that precedes the appearance of a decreased left ventricular ejection fraction [5]. Impaired LVGLS has been shown to be associated with an increased risk of HF hospitalization and mortality [6]; however, the effect of RAR on LVGLS has not yet been elucidated.

Accordingly, we aimed to assess the relationship between the RAR and subclinical myocardial impairment, as evidenced by LVGLS, in a large multi-center cohort of individuals undergoing echocardiography. In addition, we sought to further characterize the association of RAR with HF hospitalization and mortality outcomes and to determine whether these associations persisted after adjusting for clinical comorbidities and LVGLS itself.

## 2. Methods

This study was conducted in accordance with the Strengthening the Reporting of Observational Studies in Epidemiology (STROBE) guidelines [7]. It was approved by the Mayo Clinic Institutional Review Board (# 25-014428).

### 2.1. Study Population and Exposure Assessment

This retrospective cohort study included all adults over the age of 18 who underwent transthoracic echocardiography (TTE) with assessment of LVGLS across three Mayo Clinic centers (Rochester, MN, USA; Phoenix, AZ, USA; and Jacksonville, FL, USA) between January 2017 and January 2024. Eligible patients were required to have both RDW and serum albumin measured on the same calendar day, and these laboratory measurements had to occur within seven days of the index TTE to allow calculation of the RAR, by dividing the RDW by serum albumin. Our exposure of interest, the RAR, was analyzed both as a categorical variable and a continuous variable, and categorization was carried out using three tertiles based on its distribution in the study population. Clinical, laboratory, and echocardiographic data were extracted from Electronic Medical Records (EMRs) using the Mayo Data Explorer (MDE), a self-service data exploration and retrieval tool.

### 2.2. Echocardiographic Assessment

TTEs were performed using standard clinical protocols [8]. LVGLS was measured with two-dimensional speckle-tracking echocardiography using routine apical two-, three-, and four-chamber views. LVGLS was calculated as the average peak systolic longitudinal strain of all left ventricular segments and expressed as a negative percentage, where less negative values indicate worse systolic function. It is important to note that the LVGLS values used in this analysis were extracted from the MDE, and these measurements were performed by expert cardiologists as part of routine clinical follow-up for the patients. No strain measurements were conducted specifically for this research study, ensuring that the data reflect the standard clinical practice and monitoring protocols.

### 2.3. Outcome Ascertainment

The primary outcome of this study was the presence of subclinical myocardial impairment, measured by LVGLS. LVGLS was evaluated as a continuous variable and as a categorical measure using a threshold of >−18% to define abnormal strain [9]. Secondary time-to-event outcomes included all-cause mortality and acute hospitalization for HF. Mortality was ascertained from the EMRs and linked institutional files. Acute HF hospitalizations were identified using International Classification of Diseases (ICD-9/ICD-10) diagnosis codes for heart failure from inpatient encounters. For time-to-event analysis, patients were followed from the date of the index echocardiogram until the occurrence of the first outcome of interest, 5 years from the date of the index echocardiogram, last clinical follow-up, or the end of the study period (1 January 2024), whichever came first. Median follow-up was estimated using the reverse Kaplan–Meier method.

### 2.4. Covariate Assessment

Baseline clinical characteristics were obtained from the EMRs at the time of the index echocardiogram. This included age, sex, history of hypertension, diabetes mellitus, atherosclerotic CVD (in the form of coronary artery disease, peripheral arterial disease, coronary vessel revascularization, transient ischemic attacks, and previous ischemic strokes), atrial fibrillation, chronic kidney disease, and history of lipid lowering therapies (LLTs) including statin and ezetimibe use. Covariates for the multivariable models were pre-defined based on clinical relevance, biological plausibility, and established prognostic factors identified in the prior literature, rather than relying solely on statistical significance in univariable analysis.

### 2.5. Statistical Analysis

Continuous variables are presented as mean ± standard deviation or median (interquartile range), according to normality of distribution, and categorical variables as counts and percentages. Baseline characteristics were compared across RAR tertiles using Kruskal–Wallis tests for continuous variables and chi-square tests for categorical variables. The association between RAR and LVGLS was assessed using logistic regression models. The associations of RAR, as a categorical and continuous variable, with all-cause mortality and HF hospitalization were evaluated using Kaplan–Meier curves and Cox proportional hazards models. Multivariable models were adjusted for age, sex, and clinically relevant comorbidities. To account for death as a competing risk for HF hospitalization, we additionally performed Fine-Gray subdistribution hazard regression, reporting adjusted subdistribution hazard ratios (aSHR). Subgroup analyses stratified by LVGLS levels, with formal interaction testing, were also performed to further assess the robustness of these potential associations. Moreover, we compared RAR to its individual components (RDW and albumin), modeled separately, and assessed discrimination using Harrell’s C-statistic (Cox models) and the area under the receiver operating characteristic curve (AUC, logistic model) before and after adding RDW, albumin, or RAR to a base model containing age, sex, and clinical comorbidities. The shape of the association between continuous RAR and the outcomes (HF hospitalization and all-cause mortality) was examined using restricted cubic spline Cox models with four knots placed at the 5th, 35th, 65th, and 95th percentiles of RAR (2.50, 2.95, 3.44, and 5.34). The median RAR (3.15) was used as the reference at which the hazard ratio equals 1. Models were adjusted for age, sex, hypertension, diabetes mellitus, atherosclerotic cardiovascular disease, atrial fibrillation, chronic kidney disease, and antilipidemic drug use. Non-linearity was tested using the Wald test of the nonlinear spline terms. Results are reported as odds ratios (ORs) or hazard ratios (HRs) with 95% confidence intervals (CIs). A two-sided *p*-value < 0.05 was considered statistically significant. Statistical analyses were conducted using R software (version 4.5.1; R Foundation for Statistical Computing, Vienna, Austria).

## 3. Results

### 3.1. Baseline Characteristics

We included a total of 5069 individuals, of whom 1749 were in tertile 1 (RAR < 2.93), 1646 in tertile 2 (2.93 ≤ RAR ≤ 3.48), and 1674 in tertile 3 (RAR > 3.48). Age increased across RAR tertiles (54 ± 17, 63 ± 16, and 64 ± 16 years in tertiles 1, 2, and 3, respectively; *p* < 0.001). The proportion of female patients was similar across tertiles (45%, 45%, and 44%; *p* = 0.90). Hemoglobin decreased across RAR tertiles (14.10 ± 1.60, 13.06 ± 1.90, and 11.08 ± 2.35 g/dL in tertiles 1–3; *p* < 0.001), while LVEF decreased modestly (56.22 ± 8.50%, 55.33 ± 10.42%, and 53.95 ± 11.94%; *p* = 0.001). LVEF was largely preserved across the cohort overall (mean 55.2 ± 11.0%), although 19.2% of patients had reduced LVEF (<50%). In contrast, the prevalence of diabetes mellitus increased from 6% in tertile 1 to 15% in tertile 2 and 22% in tertile 3; hypertension from 47% to 57% to 65%; atrial fibrillation from 6.3% to 12% to 14%; atherosclerotic cardiovascular disease from 13% to 21% to 24%; and chronic kidney disease from 1.5% to 3.3% to 6.2% (all *p* < 0.001). Use of LLTs was also more frequent in patients with higher RAR values (15%, 23%, and 23% across tertiles 1–3; *p* < 0.001). Baseline characteristics of the study cohort are reported in Table 1.

### 3.2. RAR and Subclinical Myocardial Impairment

In total, 39.7% of patients had abnormal LVGLS (>−18%). The prevalence of abnormal strain increased across RAR tertiles, from 26.8% in tertile 1 to 41.9% in tertile 2 and 51.1% in tertile 3 (*p* < 0.001).

Using unadjusted logistic regression with tertile 1 as the reference, patients in tertile 2 had nearly twice the odds of abnormal LVGLS (OR 1.97, 95% CI 1.71–2.28; *p* < 0.001), while those in tertile 3 had almost threefold higher odds (OR 2.85, 95% CI 2.47–3.29; *p* < 0.001). The results remained significant after adjusting for clinical comorbidities and demographic factors. Compared with tertile 1, tertile 2 had an adjusted OR of 1.74 (95% CI 1.44–2.10; *p* < 0.001) and tertile 3 had an adjusted OR of 2.30 (95% CI 1.91–2.77; *p* < 0.001, Table 2). Using logistic regression with RAR modeled as a continuous variable, a higher RAR was associated with greater odds of abnormal LVGLS. In the unadjusted model, each one unit increase in the RAR was associated with 37% higher odds of abnormal LVGLS (OR 1.37, 95% CI 1.29–1.46; *p* < 0.001). This association remained significant after adjustment for clinical comorbidities and demographic factors (adjusted OR 1.25, 95% CI 1.17–1.33; *p* < 0.001). In a sensitivity analysis further adjusting for hemoglobin and LVEF, the association between the RAR and abnormal LVGLS remained essentially unchanged (aOR 1.75, 95% CI 1.48–2.08 for tertile 2; aOR 2.30, 95% CI 1.89–2.80 for tertile 3, both *p* < 0.001, Table 3). In a further sensitivity analysis restricted to patients with available eGFR (*n* = 3466, 68.4% of the cohort), additional adjustment for eGFR did not materially change these findings (Supplementary Table S1).

### 3.3. RAR and Hospitalization for Acute HF

**Figure 1:**
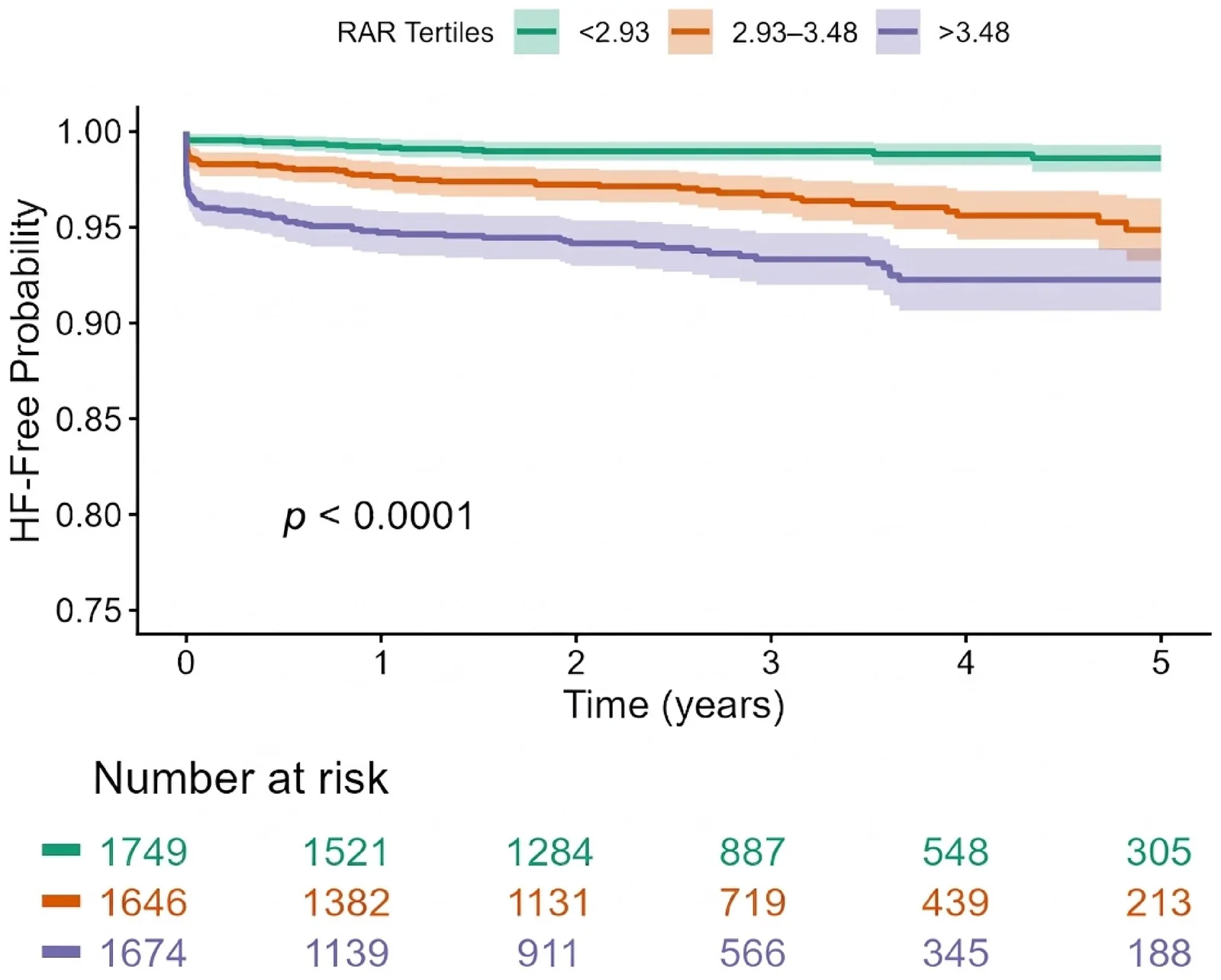
Kaplan–Meier estimates of heart failure (HF) hospitalization stratified by red cell distribution width–albumin ratio (RAR) tertiles: tertile 1 (RAR < 2.93), tertile 2 (2.93 ≤ RAR ≤ 3.48), and tertile 3 (RAR > 3.48). HF-free survival decreased stepwise across higher RAR tertiles, with the highest event rates observed in tertile 3 (log-rank *p* < 0.0001). Numbers at risk at prespecified time points are shown beneath the x-axis.

A total of 175 patients experienced HF hospitalization over a median follow-up of 2.75 years. Events and incidence rates increased across RAR tertiles, from 19 events (1.1%, 0.36 per 100 person-years) in tertile 1 to 56 events (3.4%, 1.23 per 100 person-years) in tertile 2 and 100 events (6.0%, 2.65 per 100 person-years) in tertile 3 (Table 4). Kaplan–Meier curves for HF hospitalization showed a stepwise decrease in HF-free survival across increasing RAR tertiles, with the highest event rates observed in tertile 3 (log-rank *p* < 0.0001; Figure 1). Using univariable Cox models, with tertile 1 as the reference, a higher RAR was associated with a greater risk of HF hospitalization in both tertiles 2 and 3. The HR of tertile 2 was 3.26 (95% CI 1.94–5.48; *p* < 0.001), while that of tertile 3 was almost double that of tertile 2 at 6.30 (95% CI 3.86–10.30; *p* < 0.001). Results were consistent when the RAR was measured as a continuous variable with an HR of 1.43 per one unit increase in RAR (95% CI 1.31–1.55; *p* < 0.001). After adjusting for demographic factors and clinical comorbidities, the aHR was 1.36 per one unit increase (95%CI 1.23–1.51; *p* < 0.001) with the RAR as a continuous variable, and with the RAR as a categorical variable, the aHR was 2.54 (95% CI 1.50–4.32; *p* < 0.001) for tertile 2 and 4.48 (95% CI 2.70–7.43; *p* < 0.001) for tertile 3. Additionally, in models further adjusted for abnormal LVGLS, the RAR remained independently associated with HF hospitalization. LVGLS-adjusted HRs were 2.27 (95% CI 1.33–3.85; *p* = 0.003) for tertile 2 and 3.73 (95% CI 2.24–6.20; *p* < 0.001, Table 2) for tertile 3, while the HR with the RAR as a continuous variable was 1.32 per unit increase (95% CI 1.20–1.47; *p* < 0.001, Table 2). After additional adjustment for hemoglobin and LVEF, the association between the RAR and HF hospitalization was attenuated but remained statistically significant (aHR 2.04, 95% CI 1.19–3.48, *p* = 0.009 for tertile 2; aHR 2.76, 95% CI 1.60–4.78, *p* < 0.001 for tertile 3) (Table 4). In a further sensitivity analysis restricted to patients with available eGFR (*n* = 3466, 68.4% of the cohort), additional adjustment for eGFR did not materially change these findings (Supplementary Table S1). To account for death as a competing risk for HF hospitalization, we performed a Fine-Gray subdistribution hazard model. Of 5069 patients, 175 experienced HF hospitalization and 544 died without a prior HF hospitalization (competing event). The subdistribution hazard ratios from this model (aSHR 2.49, 95% CI 1.45–4.29 for tertile 2; aSHR 4.09, 95% CI 2.42–6.93 for tertile 3; aSHR 1.31, 95% CI 1.20–1.43 per unit increase in continuous RAR) indicate that the association between the RAR and HF hospitalization persisted after accounting for the competing risk of death. Of the study cohort, 698 patients (13.8%) had prevalent HF at baseline, accounting for 32.6% of HF hospitalization events. Adding baseline HF status as a covariate did not materially change the RAR-HF hospitalization association, and the RAR remained strongly associated with incident HF hospitalization in a sensitivity analysis restricted to the 4371 patients without prevalent HF (aHR 5.01, 95% CI 2.77–9.06 for tertile 3; aHR 1.38, 95% CI 1.23–1.54 per unit increase in continuous RAR). Restricted cubic spline analysis demonstrated a significant non-linear association between the RAR and HF hospitalization (*p* for non-linearity <0.001). A non-linear association was observed, with HF hospitalization risk rising rapidly up to an RAR of approximately 3.5 and then increasing more gradually at higher RAR values (Supplementary Figure S1).

### 3.4. RAR and All-Cause Mortality

**Figure 2:**
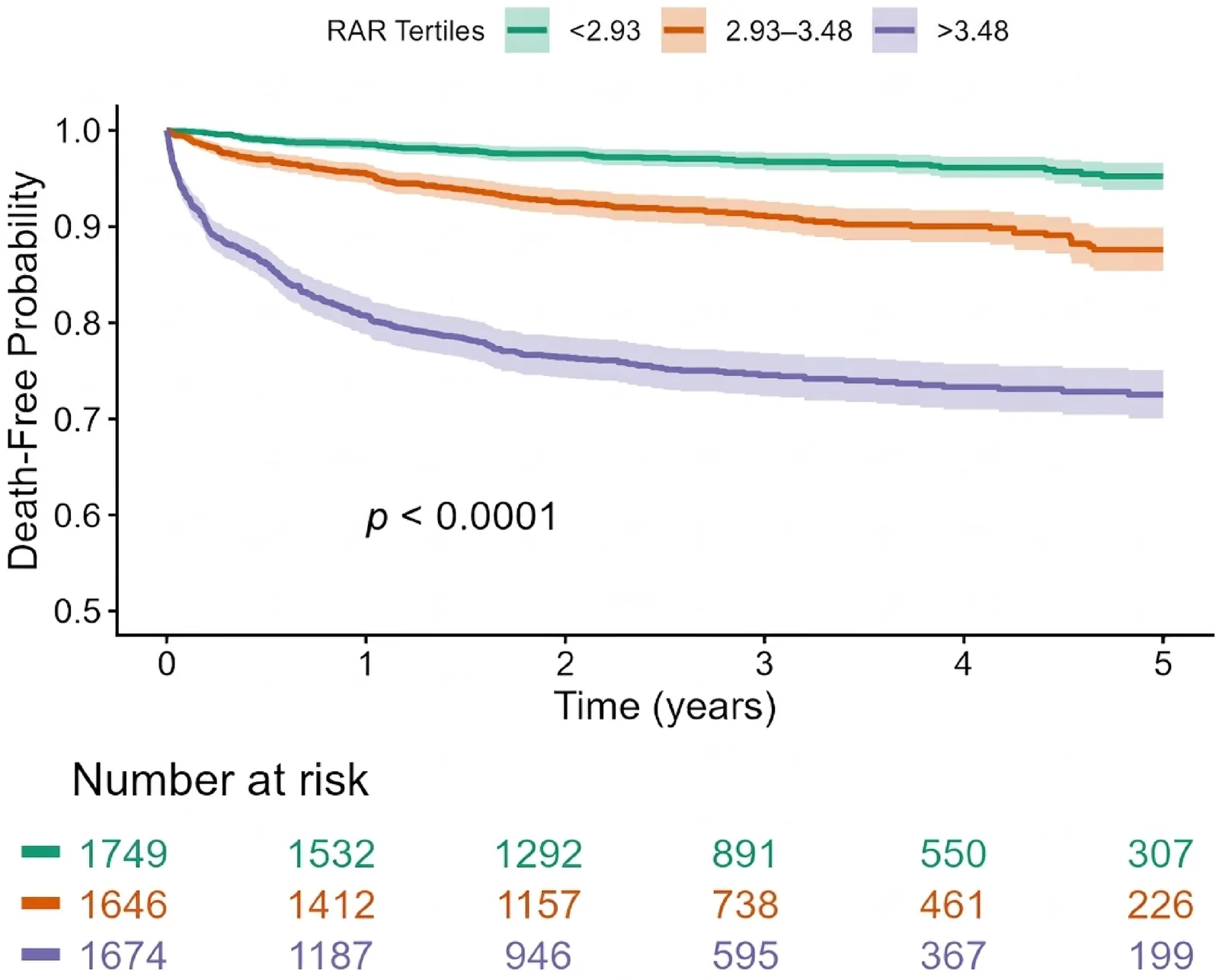
Kaplan–Meier estimates of all-cause mortality stratified by red cell distribution width–albumin ratio (RAR) tertiles: tertile 1 (RAR < 2.93), tertile 2 (2.93 ≤ RAR ≤ 3.48), and tertile 3 (RAR > 3.48). Survival decreased progressively with increasing RAR, with the highest mortality observed in tertile 3 (log-rank *p* < 0.0001). Numbers at risk at prespecified time points are shown beneath the x-axis.

**Figure 3:**
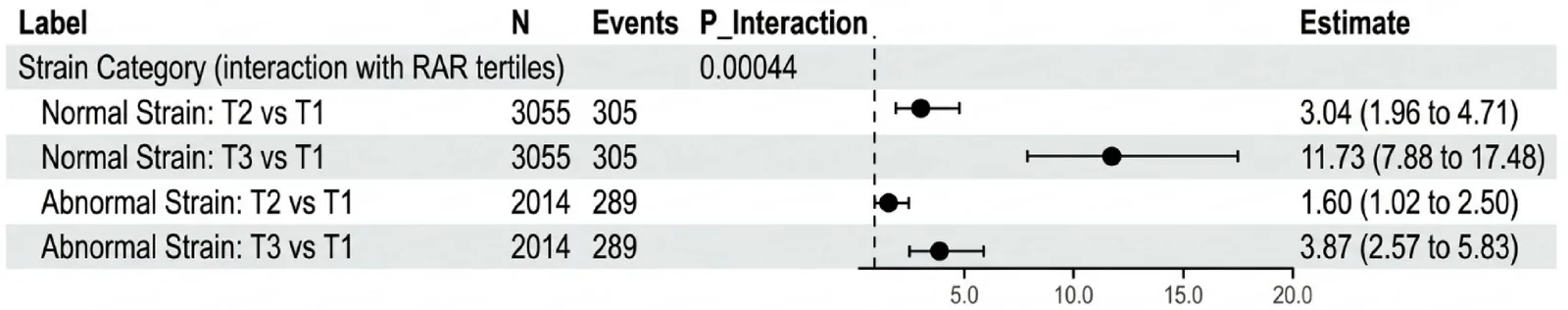
Association between RAR tertiles and all-cause mortality stratified by LVGLS status. Adjusted hazard ratios (HRs) for all-cause mortality according to red cell distribution width–albumin ratio (RAR) tertiles, stratified by left ventricular global longitudinal strain (LVGLS) status (normal vs. abnormal). Models are adjusted for age, sex, hypertension, diabetes mellitus, atherosclerotic cardiovascular disease, atrial fibrillation, chronic kidney disease, and antilipidemic drug use. Higher RAR was associated with increased mortality in both LVGLS subgroups, with larger relative effects in patients with normal LVGLS (*p* for interaction = 0.0004). Error bars indicate 95% confidence intervals (CIs). Tertile 1 (RAR < 2.93) serves as the reference category.

A total of 594 patients died over a median follow-up of 3.00 years. Events and incidence rates increased across RAR tertiles, from 56 events (3.2%, 1.06 per 100 person-years) in tertile 1 to 143 events (8.7%, 3.07 per 100 person-years) in tertile 2 and 395 events (23.6%, 10.03 per 100 person-years) in tertile 3 (Table 4). Kaplan–Meier curves for all-cause mortality showed a stepwise decrease in survival across increasing RAR tertiles, with the highest mortality observed in tertile 3 (log-rank *p* < 0.0001; Figure 2). In univariable Cox models with tertile 1 as the reference, higher RAR was strongly associated with an increased risk of death: the HR was 2.81 (95% CI 2.06–3.82; *p* < 0.001) for tertile 2 and 8.73 (95% CI 6.60–11.55; *p* < 0.001) for tertile 3. The results of RAR as a continuous variable show an HR of 1.57 (95% CI 1.51–1.63; *p* < 0.001). After adjusting for clinical comorbidities and demographic factors, the RAR remained independently associated with all-cause mortality for both continuous and categorical measurements, with aHRs of 2.39 (95% CI 1.75–3.27; *p* < 0.001) for tertile 2 and 7.30 (95% CI 5.48–9.72; *p* < 0.001, Table 2) for tertile 3, and with an aHR of 1.54 for RAR as a continuous variable (95% CI 1.48–1.60; *p* < 0.001). In an additional multivariable model that also included abnormal LVGLS, the RAR also remained strongly associated with all-cause mortality both as a continuous variable and as a categorical variable (Table 2). After additional adjustment for hemoglobin and LVEF, the association between the RAR and all-cause mortality was attenuated but remained statistically significant (aHR 1.92, 95% CI 1.40–2.64, *p* < 0.001 for tertile 2; aHR 3.94, 95% CI 2.87–5.39, *p* < 0.001 for tertile 3, Table 3). In a further sensitivity analysis restricted to patients with available eGFR (*n* = 3466, 68.4% of the cohort), additional adjustment for eGFR did not materially change these findings (Supplementary Table S1). In an interaction model including RAR tertiles and LVGLS status, LVGLS significantly modified the association between the RAR and mortality (*p* for interaction = 0.0004), with a stronger association to a higher RAR among patients with normal strain than among those with abnormal strain (Figure 3, Supplementary Table S2). Details on findings of the regression analyses are summarized in Table 2. Restricted cubic spline analysis showed a significant non-linear association between the RAR and all-cause mortality (*p* for non-linearity < 0.001). Mortality risk increased steeply as the RAR rose above approximately 3.0, with the sharpest rise through about 4.5–5.0, followed by a more gradual increase at higher RAR values (Supplementary Figure S2).

### 3.5. RAR Compared with Its Individual Components

In a supplementary analysis using the full study cohort (*n* = 5069), the RDW and albumin were each independently associated with LVGLS, HF hospitalization, and all-cause mortality (Supplementary Table S3). Discrimination improved for all three outcomes when the RDW, albumin, or the RAR were added to a base model containing age, sex, and clinical comorbidities. However, modeling the RDW and albumin as separate terms yielded discrimination equal to or better than the RAR for HF hospitalization (C-statistic 0.779 vs. 0.768) and all-cause mortality (C-statistic 0.773 vs. 0.753), while the RAR performed comparably to, and modestly better than, either component alone for LVGLS (AUC 0.720 vs. 0.718 for RDW and 0.717 for albumin) (Supplementary Table S4).

## 4. Discussion

In this large multi-center cohort of individuals who underwent echocardiography, we found that a higher RAR was associated with an increased likelihood of myocardial impairment, as evidenced by abnormal LVGLS. A higher RAR was also associated with an increased risk of acute HF hospitalization and all-cause mortality after adjustment for cardiovascular risk factors and LVGLS. Furthermore, LVGLS modified the association between the RAR and mortality, with a stronger relative effect of a higher RAR among patients with normal strain. To our knowledge, this is the first study to evaluate the association between the RAR and subclinical myocardial impairment in a general echocardiographic population and one of the few studies to investigate the RAR in a non-disease-specific cohort rather than in a single high-risk population.

The RDW reflects variation in red blood cell size and is useful in the differential diagnosis of anemia [10], yet it remains underappreciated in routine clinical practice. Elevated RDW not only indicates changes in erythropoiesis but is also associated with a wide range of pathological conditions [11]. Beyond erythrocyte disorders, several cardiovascular diseases, including HF, stroke, acute coronary syndrome, and atrial fibrillation, are frequently accompanied by changes in red blood cell size [1]. Prior studies have shown that higher RDW is positively correlated with both the incidence and mortality of cardiovascular events, which has generated increasing interest in its clinical applicability [12]. In parallel, serum albumin is a widely used negative acute phase reactant in clinical practice and is commonly applied in the assessment of malnutrition [2]. It primarily functions as a carrier protein for numerous endogenous and exogenous substances and also exhibits anti-inflammatory, antioxidant, anticoagulant, and antiplatelet aggregation properties [13,14,15]. Consistent with these roles, accumulating evidence links low serum albumin levels with the incidence of multiple cardiovascular diseases, positioning hypoalbuminemia as an important prognostic marker in these populations [15].

The RAR combines two routinely measured laboratory parameters into a single index that reflects an individual’s overall inflammatory and pathological status. Several studies have linked higher RAR values to worse outcomes in a range of cardiovascular settings, including acute myocardial infarction (AMI), atrial fibrillation, HF, and stroke. Regarding AMI, a study by Li and Xu used the MIMIC-III database to study 2081 patients and showed that those with elevated RAR had a significantly higher 90-day mortality risk (HR = 1.65, 95% CI = 1.34–2.03) [16]. For acute HF, Ni et al. observed that patients in the highest RAR quartile had consistently higher all-cause mortality at 30 days, 90 days, and one year [17]. Zhao et al. reported similar patterns in 1480 stroke patients, where the highest RAR quartile was associated with increased all-cause mortality compared with the lowest quartile [18]. In addition to its associations within cardiovascular cohorts, the RAR has also been implicated in kidney and vascular outcomes. In a retrospective cohort of 997 patients with chronic kidney disease, a higher RAR was linked to a markedly greater risk of cardiovascular events (HR = 2.27, 95% CI = 1.36–3.78; *p* < 0.001) [19], and Huang et al., in a cohort of 10,267 patients with coronary heart disease, found that an elevated RAR was significantly associated with the presence of carotid plaque (OR = 1.23, 95% CI = 1.08–1.39) [10]. Taken together, these findings support a relationship between the RAR and both the occurrence of cardiovascular diseases and the risk of cardiovascular death. Our study further adds to this literature by evaluating the RAR not only in relation to HF and mortality but also to subclinical myocardial impairment in the form of LVGLS.

The association between a higher RAR and abnormal LVGLS suggests that the RAR may reflect pathophysiologic processes that contribute to early myocardial impairment. Although the exact mechanisms underlying this relationship are not fully understood, several pathways can be proposed. Elevated RDW has been linked to chronic inflammation, oxidative stress, and impaired iron metabolism [20], all of which may promote adverse ventricular remodeling and fibrosis. Hypoalbuminemia, in turn, may indicate systemic inflammation, malnutrition, endothelial dysfunction, and altered oncotic pressure, factors that could further impact myocardial structure and function [15]. By integrating these components, a higher RAR may indicate a generally worse overall health and risk profile and help identify people in whom early myocardial damage is already present, even when the ejection fraction is still preserved. Significantly, the associations between the RAR and both HF hospitalization and all-cause mortality remained significant even after adjusting for LVGLS. This means that the association between the RAR and these outcomes is not explained by LVGLS alone. One possible explanation is that the RAR reflects systemic processes such as inflammation, nutritional status, and hematologic abnormalities that may not yet be expressed as measurable changes in LVGLS at the time of echocardiography. Furthermore, because the RAR is a combined biomarker, it may be able to capture broad multi-organ vulnerability that involves the blood, liver, and vascular function. From a clinical standpoint, the persistence of these associations even after adjusting for LVGLS suggests that the RAR may capture information not fully reflected by strain-based imaging alone. We also examined whether combining the RDW and albumin into a single ratio conferred any advantage over using these measures separately, and whether the RAR improved discrimination beyond a basic clinical model. The RDW and albumin were each significantly associated with LVGLS, HF hospitalization, and mortality, and discrimination analyses showed that modeling the RDW and albumin as separate variables performed as well as, or modestly better than, the RAR for HF hospitalization and mortality, while the RAR performed comparably to, and slightly better than, either component alone for LVGLS. These findings suggest that the value of the RAR lies primarily in its simplicity as a single, easily calculated value derived from two routinely available laboratory tests, rather than in superior statistical discrimination compared with its individual components, and that formal calibration and incremental-value analyses would be needed to establish any additional predictive utility.

Furthermore, the interaction we observed between the RAR and LVGLS status in relation to mortality helps clarify this finding. Although a higher RAR was linked to increased mortality in both the normal and abnormal strain groups, the relative impact of tertile 3 was more pronounced among individuals with normal strain compared with those with impaired strain. On an absolute scale, the RAR was associated with a 22.9 percentage-point increase in mortality risk (from 2.3% to 25.2%) across tertiles among patients with normal LVGLS, compared with a 16.3 percentage-point increase (from 5.8% to 22.1%) among those with abnormal LVGLS. This pattern suggests that the RAR is associated with mortality even among patients with preserved myocardial function on echocardiography, although it did not provide discriminative value beyond the RDW and albumin when modeled separately.

### 4.1. Strengths

This study includes a large cohort of patients who underwent echocardiography with LVGLS assessment across three Mayo Clinic sites, which improves precision and reduces dependance on single-center practice patterns. The RDW and albumin were obtained one week from index echocardiogram, making the RAR relevant to the physiological state at the time the strain was measured. A major strength is that we evaluated the RAR in relation to LVGLS, not only downstream outcomes, which adds biological plausibility to the observed associations. We also assessed clinically meaningful endpoints such as HF hospitalization and all-cause mortality using multivariable models adjusted for key demographics and comorbidities according to previous studies, and these relationships persisted even after additional adjustment for LVGLS, suggesting that these associations are not explained by strain alone. Finally, restricted cubic splines allowed us to capture non-linear relationships across the full range of the RAR.

### 4.2. Limitations

We acknowledge that our study has several limitations. First, it was a retrospective analysis from three centers within a single health system, which may limit the generalizability of our findings to other institutions or healthcare settings. Prospective, multi-center studies in more diverse populations are needed to confirm these results. Second, selection bias may be present, as we only included patients who underwent clinically indicated echocardiography with LVGLS measurement and had RDW and albumin available within seven days of the study. This likely represents a subgroup with sufficient clinical concern to warrant detailed imaging and laboratory evaluation and may not reflect truly asymptomatic community populations. Third, both the RAR and LVGLS were assessed at a single time point, so we could not evaluate how changes in these parameters over time relate to the development of subclinical myocardial dysfunction, HF, or mortality. Fourth, HF hospitalizations were identified using ICD-9/ICD-10 diagnosis codes from inpatient encounters, which may introduce some degree of misclassification, although this approach is commonly used in large observational studies [21]. Furthermore, in a supplementary analysis, the RAR did not consistently outperform albumin alone, or the RDW and albumin modeled as separate variables, in discrimination for HF hospitalization and mortality; the incremental value of combining these markers into a single ratio, rather than using them separately, therefore remains uncertain for these outcomes. Additionally, LVGLS was measured during routine clinical care across three sites over a seven-year period, involving multiple readers and likely multiple ultrasound vendors, without a central core laboratory or formal reproducibility assessment; given that GLS values can vary between vendors and software platforms, the application of a single fixed threshold (−18%) across this heterogeneous set of measurements may have introduced some degree of measurement variability or misclassification. Moreover, cause-specific mortality data (cardiovascular versus non-cardiovascular death) were not available, and given the nonspecific nature of the RAR, a substantial portion of the observed all-cause mortality may be attributable to non-cardiovascular causes; this should be considered when interpreting the mortality findings of this study. We also note that we lacked data on whether the index laboratory measurements and echocardiogram were obtained in an inpatient or outpatient setting; because albumin is a negative acute-phase reactant, values drawn during acute illness may be transiently depressed independent of baseline nutritional or inflammatory status, which could contribute to the observed association between the RAR and mortality. Finally, as with all observational analyses, residual confounding from unmeasured or incompletely measured variables cannot be excluded.

## 5. Conclusions

The RAR was associated with myocardial impairment, as reflected by abnormal LVGLS, and with an increased risk of HF hospitalization and all-cause mortality. The RAR improved discrimination over a clinical model alone but did not provide discriminative value beyond its individual components, RDW and albumin. Prospective studies incorporating formal calibration analyses are needed to confirm these results.

## Figures and Tables

**Table 1:** Baseline characteristics by RAR tertile.

Characteristic	First Tertile N = 1749 ^1^	Second Tertile N = 1646 ^1^	Third Tertile N = 1674 ^1^	*p*-Value ^2^
Age	54.07 ± 17.22	63.21 ± 16.02	64.01 ± 15.79	<0.001
Sex				0.89
Males	965 (55.2%)	901 (54.7%)	930 (55.6%)	
Females	784 (44.8%)	745 (45.3%)	744 (44.4%)	
Abnormal strain	469 (26.8%)	690 (41.9%)	855 (51.1%)	<0.001
Hemoglobin (g/dL)	14.10 ± 1.60	13.06 ± 1.90	11.08 ± 2.35	<0.001
LVEF (%)	56.22 ± 8.50	55.33 ± 10.42	53.95 ± 11.94	0.001
Type 2 DM	105 (6.0%)	239 (15%)	366 (22%)	<0.001
Hypertension	823 (47%)	934 (57%)	1084 (65%)	<0.001
Atrial fibrillation	111 (6.3%)	196 (12%)	242 (14%)	<0.001
ASCVD	230 (13%)	340 (21%)	402 (24%)	<0.001
CKD	27 (1.5%)	54 (3.3%)	104 (6.2%)	<0.001
Antilipidemic drugs	259 (15%)	372 (23%)	391/1674 (23%)	<0.001

^1^ Mean ± SD, n/N (%). ^2^ Kruskal–Wallis rank sum test; Pearson’s Chi-squared test. Type 2DM: type 2 diabetes mellitus; ASCVD: atherosclerotic cardiovascular disease; CKD: chronic kidney disease; LVEF: left ventricular ejection fraction.

**Table 2:** Association of RAR tertiles with abnormal LVGLS, acute heart failure hospitalization, and all-cause mortality.

Outcome (Model Type)	Unadjusted Estimate (95% CI); *p*	Adjusted Estimate * (95% CI); *p*	Adjusted Estimate ** (95% CI); *p*
Abnormal LVGLS (>−18%) (OR)			
Tertile 2 vs. 1	OR 1.97 (1.71–2.28); *p* < 0.001	OR 1.74 (1.44–2.10); *p* < 0.001	—
Tertile 3 vs. 1	OR 2.85 (2.47–3.29); *p* < 0.001	OR 2.30 (1.91–2.77); *p* < 0.001	—
RAR as a continuous variable	OR 1.37 (1.29–1.46); *p* < 0.001	OR 1.25 (1.17–1.33); *p* < 0.001	
Acute HF hospitalization (HR)			
Tertile 2 vs. 1	HR 3.26 (1.94–5.48); *p* < 0.001	HR 2.54 (1.49–4.32); *p* < 0.001	HR 2.27 (1.33–3.85); *p* = 0.003
Tertile 3 vs. 1	HR 6.30 (3.86–10.30); *p* < 0.001	HR 4.48 (2.70–7.43); *p* < 0.001	HR 3.73 (2.24–6.20); *p* < 0.001
RAR as a continuous variable	HR 1.43 (1.31–1.55); *p* < 0.001	HR 1.36 (1.23–1.51); *p* < 0.001	HR 1.32 (1.20–1.47); *p* < 0.001
All-cause mortality (overall) (HR)			
Tertile 2 vs. 1	HR 2.81 (2.06–3.82); *p* < 0.001	HR 2.39 (1.75–3.27); *p* < 0.001	HR 2.41 (1.76–3.30); *p* < 0.001
Tertile 3 vs. 1	HR 8.73 (6.60–11.55); *p* < 0.001	HR 7.30 (5.48–9.72); *p* < 0.001	HR 7.38 (5.53–9.84); *p* < 0.001
RAR as a continuous variable	HR 1.57 (1.51–1.63); *p* < 0.001	HR 1.54 (1.48–1.60); *p* < 0.001	HR 1.54 (1.48–1.60); *p* < 0.001
All-cause mortality, normal LVGLS (HR)			
Tertile 2 vs. 1	—	HR 3.04 (1.96–4.72); *p* < 0.001	—
Tertile 3 vs. 1	—	HR 11.73 (7.88–17.48); *p* < 0.001	—
All-cause mortality, abnormal LVGLS (HR)			
Tertile 2 vs. 1	—	HR 1.60 (1.02–2.50); *p* = 0.039	—
Tertile 3 vs. 1	—	HR 3.87 (2.57–5.83); *p* < 0.001	—

* Adjusted for age, sex, hypertension, diabetes mellitus, atherosclerotic cardiovascular disease, atrial fibrillation, chronic kidney disease, and use of antilipidemic drugs. ** Additionally adjusted for abnormal LVGLS. Unadjusted models are univariable models with RAR tertiles as the only predictor.

**Table 3:** Association of RAR tertiles with study outcomes, before and after adjustment for hemoglobin and LVEF.

Outcome	Original Adjusted Estimate (95% CI); *p*	Hb/EF-Adjusted Estimate (95% CI); *p*
LVGLS (OR), Tertile 2 vs. 1	1.74 (1.44–2.10); *p* < 0.001	1.75 (1.48–2.08); *p* < 0.001
LVGLS (OR), Tertile 3 vs. 1	2.30 (1.91–2.77); *p* < 0.001	2.30 (1.89–2.80); *p* < 0.001
HF hospitalization (HR), Tertile 2 vs. 1	2.54 (1.49–4.32); *p* < 0.001	2.04 (1.19–3.48); *p* = 0.009
HF hospitalization (HR), Tertile 3 vs. 1	4.48 (2.70–7.43); *p* < 0.001	2.76 (1.60–4.78); *p* < 0.001
All-cause mortality (HR), Tertile 2 vs. 1	2.39 (1.75–3.27); *p* < 0.001	1.92 (1.40–2.64); *p* < 0.001
All-cause mortality (HR), Tertile 3 vs. 1	7.30 (5.48–9.72); *p* < 0.001	3.94 (2.87–5.39); *p* < 0.001

**Table 4:** Event counts, follow-up, and incidence rates by RAR tertile.

RAR Tertile	N	HF Events	HF %	HF Person-Years	HF IR/100 Person-Years	Death Events	Death, %	Death Person-Years	Death IR/100 Person-Years
1 (<2.93)	1749	19	1.1%	5233.7	0.36	56	3.2%	5269.3	1.06
2 (2.93–3.48)	1646	56	3.4%	4541.6	1.23	143	8.7%	4662.4	3.07
3 (>3.48)	1674	100	6.0%	3771.8	2.65	395	23.6%	3937.0	10.03

## Data Availability

The data underlying this article will be shared upon reasonable request to the corresponding author.

## Supplementary Materials

The following supporting information can be downloaded at: https://www.mdpi.com/article/10.3390/jpm16100530/s1, Figure S1: Restricted Cubic Spline Analysis of the Association Between RAR and Heart Failure Hospitalization Figure S2: Restricted Cubic Spline Analysis of the Association Between RAR and All-Cause Mortality. Table S1. GFR sensitivity, n = 3466 with eGFR available. Table S2. Deaths by RAR tertile within each LVGLS stratum. Table S3. Association of RDW, albumin, and RAR (continuous) with study outcomes. Table S4. Discrimination of a base clinical model with and without RDW, albumin, or RAR.
