# Supplementary material for: Association of the Red Cell Distribution Width–Albumin Ratio with Myocardial Strain, Mortality, and Heart Failure Hospitalization: A Retrospective Cohort Study
Source: J Pers Med. 2026 Oct 14;16(10):530. doi: 10.3390/jpm16100530 (PMC13706101; doi:10.3390/jpm16100530)
Supplement: Supplementary file 1 [file jpm-16-00530-s001.zip › Supplementary Table S1.pdf]

Supplementary Table S1. GFR sensitivity, n=3,466 with eGFR available

Outcome	Hb/EF-adjusted	Hb/EF/GFR-adjusted
Mortality, continuous	1.31 (1.24–1.38)	1.26 (1.19–1.34)
Mortality, T2 vs 1	1.92 (1.40–2.64)	1.90 (1.31–2.75)
Mortality, T3 vs 1	3.94 (2.87–5.39)	4.23 (2.94–6.10)
HF hosp., continuous	1.22 (1.07–1.39)	1.17 (1.02–1.34)
HF hosp., T2 vs 1	2.04 (1.19–3.48)	2.06 (1.15–3.68)
HF hosp., T3 vs 1	2.76 (1.60–4.78)	3.02 (1.67–5.48)
LVGLS, continuous	1.22 (1.13–1.32)	1.28 (1.17–1.42)
LVGLS, T2 vs 1	1.75 (1.48–2.08)	1.51 (1.23–1.86)
LVGLS, T3 vs 1	2.30 (1.89–2.80)	2.23 (1.75–2.85)

eGFR was available in 3,466 of 5,069 patients (68.4%); this sensitivity analysis is restricted to patients with complete data on hemoglobin, LVEF, and eGFR
