# Supplementary material for: Association of the Red Cell Distribution Width–Albumin Ratio with Myocardial Strain, Mortality, and Heart Failure Hospitalization: A Retrospective Cohort Study
Source: J Pers Med. 2026 Oct 14;16(10):530. doi: 10.3390/jpm16100530 (PMC13706101; doi:10.3390/jpm16100530)
Supplement: Supplementary file 1 [file jpm-16-00530-s001.zip › Supplementary Table S2.pdf]

Supplementary Table S2. Deaths by RAR tertile within each LVGLS stratum

**LVGLS status**	**Tertile**	**N**	**Deaths**	**Death %**
Normal	1	1280	29	2.3%
Normal	2	956	70	7.3%
Normal	3	819	206	25.2%
Abnormal	1	469	27	5.8%
Abnormal	2	690	73	10.6%
Abnormal	3	855	189	22.1%
