# Supplementary material for: Association of the Red Cell Distribution Width–Albumin Ratio with Myocardial Strain, Mortality, and Heart Failure Hospitalization: A Retrospective Cohort Study
Source: J Pers Med. 2026 Oct 14;16(10):530. doi: 10.3390/jpm16100530 (PMC13706101; doi:10.3390/jpm16100530)
Supplement: Supplementary file 1 [file jpm-16-00530-s001.zip › Supplementary Table S3.pdf]

Supplementary Table S3. Association of RDW, albumin, and RAR (continuous) with study outcomes

Outcome	Predictor	Estimate	95% CI	p-value
Mortality (HR)	RDW alone	1.194	1.167–1.222	<0.001
Mortality (HR)	Albumin alone	0.340	0.306–0.378	<0.001
Mortality (HR)	RAR	1.541	1.481–1.603	<0.001
HF hospitalization (HR)	RDW alone	1.122	1.064–1.183	<0.001
HF hospitalization (HR)	Albumin alone	0.514	0.413–0.639	<0.001
HF hospitalization (HR)	RAR	1.363	1.233–1.507	<0.001
LVGLS (OR)	RDW alone	1.109	1.077–1.142	<0.001
LVGLS (OR)	Albumin alone	0.691	0.620–0.769	<0.001
LVGLS (OR)	RAR	1.249	1.174–1.331	<0.001
