# Supplementary material for: Association of the Red Cell Distribution Width–Albumin Ratio with Myocardial Strain, Mortality, and Heart Failure Hospitalization: A Retrospective Cohort Study
Source: J Pers Med. 2026 Oct 14;16(10):530. doi: 10.3390/jpm16100530 (PMC13706101; doi:10.3390/jpm16100530)
Supplement: Supplementary file 1 [file jpm-16-00530-s001.zip › Supplementary Table S4.pdf]

Supplementary Table S4. Discrimination of a base clinical model with and without RDW, albumin, or RAR

Outcome	Model	C-statistic / AUC
**Mortality (C-statistic)**	**Base (age, sex, comorbidities)**	**0.6251**
**Mortality (C-statistic)**	**Base + RDW**	**0.7122**
**Mortality (C-statistic)**	**Base + Albumin**	**0.7541**
**Mortality (C-statistic)**	**Base + RDW + Albumin**	**0.7726**
**Mortality (C-statistic)**	**Base + RAR**	**0.7525**
**HF hospitalization (C-statistic)**	**Base (age, sex, comorbidities)**	**0.7340**
**HF hospitalization (C-statistic)**	**Base + RDW**	**0.7506**
**HF hospitalization (C-statistic)**	**Base + Albumin**	**0.7764**
**HF hospitalization (C-statistic)**	**Base + RDW + Albumin**	**0.7794**
**HF hospitalization (C-statistic)**	**Base + RAR**	**0.7681**
**LVGLS (AUC)**	**Base (age, sex, comorbidities)**	**0.7073**
**LVGLS (AUC)**	**Base + RDW**	**0.7176**
**LVGLS (AUC)**	**Base + Albumin**	**0.7174**
**LVGLS (AUC)**	**Base + RDW + Albumin**	**0.7215**
**LVGLS (AUC)**	**Base + RAR**	**0.7196**
